# Electron paramagnetic resonance microscopy using spins in diamond under ambient conditions

**DOI:** 10.1038/s41467-017-00466-y

**Published:** 2017-09-06

**Authors:** David A. Simpson, Robert G. Ryan, Liam T. Hall, Evgeniy Panchenko, Simon C. Drew, Steven Petrou, Paul S. Donnelly, Paul Mulvaney, Lloyd C. L. Hollenberg

**Affiliations:** 1 School of Physics, University of Melbourne, Parkville, VIC 3010 Australia; 2 Centre for Neural Engineering, University of Melbourne, Parkville, VIC 3010 Australia; 3 School of Chemistry and Bio21 Molecular Science and Biotechnology Institute, The University of Melbourne, Parkville, VIC 3010 Australia; 4 Department of Medicine, Royal Melbourne Hospital, The University of Melbourne, Parkville, VIC 3010 Australia; 5 Florey Neuroscience Institute, University of Melbourne, Parkville, VIC 3010 Australia; 6 Centre for Integrated Brain Function, University of Melbourne, Parkville, VIC 3010 Australia; 7 Centre for Quantum Computation and Communication Technology, University of Melbourne, Parkville, VIC 3010 Australia

## Abstract

Magnetic resonance spectroscopy is one of the most important tools in chemical and bio-medical research. However, sensitivity limitations typically restrict imaging resolution to ~ 10 µm. Here we bring quantum control to the detection of chemical systems to demonstrate high-resolution electron spin imaging using the quantum properties of an array of nitrogen-vacancy centres in diamond. Our electron paramagnetic resonance microscope selectively images electronic spin species by precisely tuning a magnetic field to bring the quantum probes into resonance with the external target spins. This provides diffraction limited spatial resolution of the target spin species over a field of view of 50 × 50 µm^2^ with a spin sensitivity of 10^4^ spins per voxel or ∼100 zmol. The ability to perform spectroscopy and dynamically monitor spin-dependent redox reactions at these scales enables the development of electron spin resonance and zepto-chemistry in the physical and life sciences.

## Abstract (web summary)

Electron paramagnetic resonance spectroscopy has important scientific and medical uses but improving the resolution of conventional methods requires cryogenic, vacuum environments. Simpson et al. show nitrogen vacancy centres can be used for sub-micronmetre imaging with improved sensitivity in ambient conditions.

## Introduction

Magnetic resonance spectroscopy techniques have revolutionised detection and imaging capabilities across the life and physical sciences. Electron paramagnetic resonance (EPR), nuclear magnetic resonance (NMR), and magnetic resonance imaging (MRI) are now essential tools in many areas of science and clinical research. Current ambient NMR^1^ and EPR-based systems^2^ employing field gradients have demonstrated imaging resolution as low as 10 µm. However, exploring nanoscale biological and chemical processes with sub-micrometre resolution requires a major technological shift.

State-of-the-art cryogenic EPR-based imaging approaches have demonstrated detection from 10^6^ spins at sub-micrometre spatial resolution, by reducing the size of the surface loop and scanning, with projected sensitivity down to the 10–100 spins regime^3^. Other high-resolution imaging techniques such as magnetic resonance force microscopy^4^ and scanning tunnelling microscopy provide single-electron spin sensitivity with nanoscale spatial resolution^5^, but they are also constrained to cryogenic temperatures and high vacuum environments which precludes their use in imaging functional biochemical reactions. For EPR applications, the regime of sub-micrometre room temperature spectroscopy and imaging has presented a major challenge. The development of such a technology would provide insights into electron spin dynamics at the nanoscale, including redox dynamics, organic radical formation and complicated transition metal biochemistry at the intra-cellular scale.

**Fig. 1:**
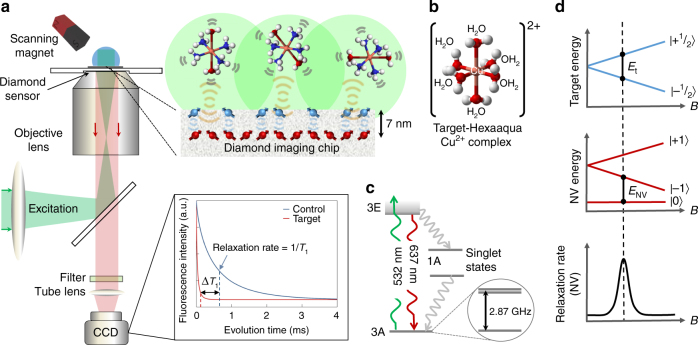
Quantum probe relaxation microscopy. **a** Schematic of the microscope system with optical excitation at 532 nm and resulting NV fluorescence filtered (650–750 nm) and imaged onto an sCMOS camera. Microwave control is provided via a gold microwave resonator evaporated onto a glass coverslip. Expanded view shows the diamond imaging chip with target and surface electronic spins, in addition to the layer of NV centres 7 nm below the surface. The spin lattice relaxation time, *T*_1_, across the imaging array is determined from a sequence of fluorescence images, see Fig. 2 for more details. **b** Schematic representation of the spin target hexaaqua-Cu^2+^ complex, [Cu(OH_2_)_6_]^2+^. **c** Simplified energy-level diagram for the NV centre highlighting the paramagnetic ground-state triplet. At zero magnetic field the degenerate |−1〉 and |+1〉 states are separated from the |0〉 state by the crystal field splitting of *D* = 2.87 GHz. **d** Quantum probe relaxation spectroscopy. Simplified energy diagrams representing the Zeeman splitting for a target spin (Cu^2+^) and the NV probe. When the transition energy, *E*_t_, from the target spin (|−^1^/_2_〉 → |+^1^/_2_〉) is matched to the transition energy, *E*_NV_, from the NV centre (|0〉 → |−1〉), the NV spin relaxation rate (1/*T*_1_) signal increases

Here we report an imaging technique based on quantum probe relaxation (QPR) spectroscopy, which can selectively image spectrally resolved spin targets in aqueous solution with high spatial resolution under ambient conditions. Electron spin detection is achieved using an array of nitrogen vacancy (NV) spin probes in diamond together with wide-field optical microscopy and precise magnetic field alignment as shown in Fig. 1a. As a demonstration of the capabilities, we perform spectroscopic imaging of hexaaqua-Cu^2+^ complexes, see Fig. 1b, and their associated redox dynamics over fields of view (FOV) of ~ 50 × 50 µm^2^. We demonstrate imaging resolution at the diffraction limit (~ 300 nm) with spin sensitivities in the hundreds of zeptomol (10^−21^ mol) range.

**Fig. 2:**
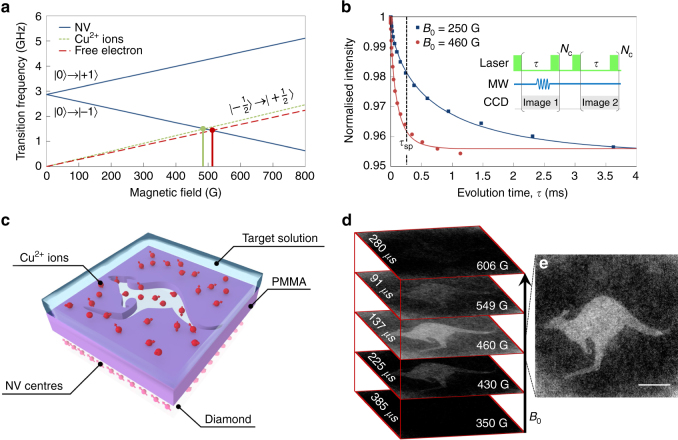
Quantum probe relaxation imaging of aqueous Cu^2+^. **a** Transition frequencies of the hexaaqua-Cu^2+^ ions and free electrons (|−^1^/_2_ 〉 → |+^1^/_2_〉) and NV spin probes (|0〉 → |−1〉 and |0〉 → |+1〉) as a function of the applied B-field. The solid *green line* depicts the resonant position of NV and Cu^2+^ (487 G), while the solid *red line* shows the resonance condition for NV and free electron spins (512 G). **b** *T*_1_ relaxation curves measured ‘near’ (460 ± 2 G) and ‘off’ (250 ± 2 G) resonance with Cu^2+^ ions. *Inset*: *T*_1_ measurement sequence. Measurements were averaged over *N*_c_ *=* 10,000 cycles at each ‘*τ*’ point, with a typical acquisition time of 5 min. **c** Schematic of the PMMA image template on the diamond imaging chip. **d** Single *τ*_sp_ images at various magnetic fields. The measurement sequence for the single *τ*_sp_ images is the same as in **b** with *τ* = *τ*_sp_. The probe time, *τ*_sp_, at each magnetic field is shown on the left-hand side of the image. Images were averaged over *N*_c_ *=* 6 × 10^6^ cycles. The total acquisition time per image was 40 min. **e** Quantum probe relaxation image showing the spatial distribution of Cu^2+^ ions near the Cu^2+^ ↔ NV resonance at 460 G. The *scale bar* is 10 µm

## Results

### EPR with NV centres in diamond

Electron paramagnetic resonance microscopy is performed by monitoring the spin lattice relaxation times of an array of solid state atomic sized quantum sensors in diamond. The NV probes are engineered at controlled depths (~ 6–8 nm) from the diamond surface with an average spatial separation of ~ 10 nm via nitrogen ion-implantation (see ‘Methods’). Due to the crystal symmetry of diamond, the quantum probes are formed along four possible 〈111〉 crystallographic orientations. The NV centre in diamond has a paramagnetic ground-state triplet as shown in Fig. 1c with the |±1〉 states separated from the |0〉 state by the diamond zero-field splitting *D* *=* 2.87 GHz. The degeneracy of the |±1〉 states is lifted in the presence of a magnetic field and Zeeman split with a gyromagnetic ratio *γ*_NV_ = 2.8 MHz G^−1^. The quantum probes can be conveniently spin polarised and their state ʽreadout’ at room temperature under optical excitation at 532 nm. The spin state readout arises from the difference in fluorescence intensity between the |0〉 and |±1〉 states, allowing optically detected magnetic resonance (ODMR) of the ground-state magnetic sublevels^6^. In the case of NV ensembles, the four orientations result in eight possible transitions depending on the applied magnetic field. An external field can be applied along a particular NV symmetry axis and a single microwave *π* pulse used to isolate the subset of NV spin probes which are aligned with the applied magnetic field. The spin lattice relaxation time (*T*_1_) of the quantum probes can be determined by optically polarising the spins into the *m*_s_ = 0 ground state, then allowing them to evolve for a variable time *τ*, before sampling the spin population with an additional optical pulse. Interactions between the NV probe and nearby electronic, nuclear and surface spins species cause the NV net magnetisation to relax from the *m*_s_ = 0 state to a mixture of the three ground triplet states. The 1/*e* decay time is referred to as the *T*_1_ time of the probe.

The NV centre^7^ in diamond is a promising system for nanoscale electronic spin detection ideally suited for room temperature biological environments^8–10^. While detection of small numbers of electronic spins (less than 10), based on decoherence^11^, or quantum control protocols^12, 13^ has been carried out using single NV centres^13–17^, and in wide-field array systems^18, 19^, spectroscopic microscopy that can target specific electronic spin species is lacking. Spectroscopic detection to date has relied on complex quantum control techniques^20, 21^, which creates major challenges when scaling up to large FOV. The main challenge here is creating a uniform driving field over a wide FOV to address the NV probes and target spins. We instead employ an optical spectroscopic detection method^22^ based on precise control of a static external magnetic field, *B*_0_, which is used to tune the NV transition energy into resonance with environmental spins at selected effective *g*-factors. At a given resonance condition the NV and environmental spins (which may comprise intrinsic and target spins) exchange energy resulting in an increase in the measured NV relaxation rate as illustrated in Fig. 1d. This quantum probe relaxation microscopy (QPRM) allows the EPR spectrum of the target spins to be resolved at each imaging pixel.

The relaxation rate, ${\rm{\Gamma }}_1^{\left( {\rm{E}} \right)}\left( {{B_0}} \right) = 1/{T_1}$, of a quantum probe in the presence of an arbitrary magnetic environment (E), comprising intrinsic (I) and/or target (T) spins, is given by the spectral density of the environment *S* ^(E)^(*ω*_E_, *B*_0_) convolved with the filter function of the probe, in this case the NV spin:

1 $${\rm{\Gamma }}_1^{\left( {\rm{E}} \right)}\left( {{B_0}} \right) = \mathop {\int }{S^{\left( {\rm{E}} \right)}}\left( {{\omega _{\rm{E}}},{B_0}} \right)G\left( {{{\rm{\Gamma }}_2},{\omega _{\rm{E}}},{B_0}} \right)\,{\rm{d}}{\omega _{\rm{E}}},$$

where *G*(Γ_2_, *ω*_E_, *B*_0_)$= \frac{{{b^2}{\Gamma _2}}}{{2\left( {{\rm{\Gamma }}_2^2 + {{\left( {{\omega _{{\rm{NV}}}} - {\omega _{\rm{E}}}} \right)}^2}} \right)}}$ is the NV filter function given by a Lorentzian dependent on the resonance frequency of environmental spins, *ω*_E_, the coupling strength, *b*, the external magnetic field *B*_0_(*ω*_NV_ = *γ*_NV_ *B*_0_) and NV transverse relaxation rate Γ_2_ (Supplementary Note 1). To probe specific components of the environment’s spectral density, *S* ^(E)^(*ω*_E_, *B*_0_), the NV filter function can be tuned via the Zeeman interaction and an applied magnetic field, *B*_0_.

To characterise the inherent magnetic environment of the diamond sensing chip, a calibration experiment is carried out to obtain the relaxation rate spectrum,${\rm{\Gamma }}_1^{\left( {\rm{I}} \right)}\left( {{B_0}} \right)$ in the absence of a target system. The calibration spectrum reflects the interactions of electronic and nuclear spins both on the surface and within the bulk diamond. The target (T) is then introduced and the NV relaxation rate spectrum corresponding to the combined system, ${\rm{\Gamma }}_1^{\left( {{\rm{I + T}}} \right)}\left( {{B_0}} \right)$, mapped. By subtracting the calibration measurement, we obtain the target relaxation spectrum below, see Supplementary Note 1 for details:

2 $${\rm{\Gamma }}_1^{\left( {\rm{T}} \right)}\left( {{B_0}} \right) \approx {\rm{\Gamma }}_1^{\left( {{\rm{I}} + {\rm{T}}} \right)}\left( {{B_0}} \right) - {\rm{\Gamma }}_1^{\left( {\rm{I}} \right)}\left( {{B_0}} \right),$$

Given the relatively narrow probe filter function, the target relaxation spectrum is essentially unchanged following deconvolution of Eq. (1) and is therefore identical to the spectral density *S* ^(T)^(*ω*_E_; *B*_0_), up to a normalisation factor.

### QPR imaging of hexaaqua-Cu^2+^ ions

Copper reactions are tightly regulated in biology due to the potential for free copper ions to produce damaging free radical species^23–25^. Therefore, there is significant interest in understanding how Cu^2+^ and similar transition metal ions interact in varying chemical environments. To date, the majority of techniques used to characterise Cu^2+^ ions in biological systems have involved measuring luminescent properties of Cu^2+^-bound molecular probes^26, 27^. Redox reactions of Cu^2+^ and other transition metals in solution have traditionally been studied using electro-chemical approaches and fluorescent molecular probes that act as surrogate markers of redox status^28^. The QPRM approach described here is label free and non-invasive and does not impact on the function or availability of the Cu^2+^ complexes. Using spin selective spectroscopy, we can probe redox reaction kinetics in sensing volumes of order attoliters with temporal resolution of order seconds enabling the study of transition metal ions in solution and/or bulk material systems.

We begin with the demonstration of selective detection and spatial imaging of Cu^2+^ ions. Figure 2a shows the transition frequencies of the NV probe, free electron spins (〈*g*〉 = 2) and the target hexaaqua Cu^2+^ ion (〈*g*_eff_〉 = 2.199) as a function of magnetic field. The electronic configuration of the target spin complex [Cu(OH_2_)_6_]^2+^, is *d* ^9^ with octahedral geometry, giving rise to a single unpaired electron, and an overall electronic spin of ½. Degeneracy resulting from Jahn–Teller distortion, see Fig. 1b, gives rise to an axially symmetric Zeeman interaction with a perpendicular and parallel *g*-factor of *g*_⊥_ = 2.099 and *g*_‖_ = 2.400 ^29^, respectively. At room temperature, a weighted average of these two *g* factors is observed due to motional averaging; when integrated over all possible orientations this results in an isotropic *g*-factor of 〈*g*〉 = 1/3(*g*_‖_ + 2 *g*_⊥_) = 2.199. From Fig. 2a the resonance condition for Cu^2+^ ions ↔ NV occurs around 487 G. At the resonance point the Cu^2+^ and NV probe spins can exchange energy efficiently resulting in a significant reduction in the NV *T*_1_ time (increase in the relaxation rate), forming the fundamental QPRM image contrast.

Figure 2b presents two *T*_1_ relaxation curves (measurement sequence shown in the inset) off (250 ± 1 G) and near resonance (460 ± 2 G) with Cu^2+^ spins. The *T*_1_ time from the NV probes reduced from 730 ± 60 to 96 ± 1 µs as the Cu^2+^ resonance condition was approached. This reduction included a contribution from surface spins, the analysis and subtraction of which is described quantitatively in the next section on spectroscopy. To image the Cu^2+^ distribution in solution an image mask was fabricated on top of the diamond imaging chip with poly(methyl methacrylate) (PMMA) as described in Fig. 2c (see ‘Methods’). A set of single evolution time point, *τ*_sp_, images were obtained at magnetic fields either side of the Cu^2+^ ↔ NV resonance position. The *τ*_sp_ probe time was set to the *T*_1_ time obtained from the FOV at each respective magnetic field. This probe time maximised image contrast for small signal changes. The single *τ*_sp_ images were averaged over *N*_c_ = 6 × 10^6^ cycles and are shown in Fig. 2d.

The QPR image shown in Fig. 2e demonstrates significant image contrast from an effective 2D distribution of Cu^2+^ ions defined by the image mask at magnetic fields near the Cu^2+^ ↔ NV resonance condition. This form of imaging is sufficient to spatially image the Cu^2+^ distribution in solution. In the following section, we perform quantitative spectroscopy on these target spins.

### Quantitative electronic spin spectroscopy

**Fig. 3:**
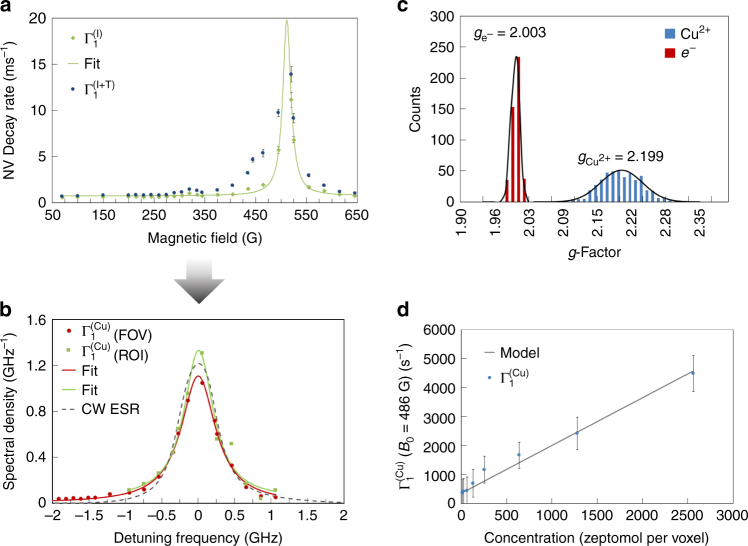
High-resolution electronic paramagnetic resonance imaging and spectroscopy. **a** *T*_1_ decay rate as a function of applied magnetic field *B*_0_ for 1 mM of nitric acid, (intrinsic-*green diamonds*) and 100 mM of Cu^2+^ ions, (intrinsic + target-*blue circles*). The uncertainty in the relaxation is given by the rms error on the *T*_1_ stretched exponential fit. **b** Relaxation rate spectra, ${\rm{\Gamma }}_1^{\left( {{\rm{Cu}}} \right)}$, identified by subtracting $\Gamma _1^{\left( {{\rm{I}} + {\rm{T}}} \right)} - {\rm{\Gamma }}_1^{\left( {\rm{I}} \right)}$ from the field of view (*FOV*) (35 × 35 µm^2^) and region of interest (*ROI*) (1.6 × 1.6 µm^2^). The absorption peak at 486 ± 1 G corresponds to a *g*-factor of 2.21 ± 0.02, consistent with the effective *g*-factor for aqueous Cu^2+^ ions from conventional CW EPR, see *dashed grey line*. **c** Histogram of the measured *g*-factor from each imaging voxel for Cu^2+^ ions and free electrons. The distribution of *g*-factors for the free electrons is centred at *g* = 2.003 with a standard deviation of 0.007, while the Cu^2+^ distribution exhibits a distribution centred at *g* = 2.199 with a standard deviation of 0.03. **d** Measured relaxation rate, ${\rm{\Gamma }}_1^{\left( {{\rm{Cu}}} \right)}$, on resonance (*B*_0_ = 486 G) vs. Cu^2+^ concentration. The *error bars* are given by the rms error on the *T*_1_ stretched exponential fit. The minimum detectable number of spins in a sensing volume defined by 0.025 µm^3^ or (25 aL) is ~ 75,000 spins per voxel or ~ 125 zmol per voxel

To demonstrate quantitative spectroscopy, a thorough understanding of the intrinsic magnetic environment consisting of the surface and intrinsic spins is required before introducing external target spins into the environment. Calibration measurements were conducted with a solution of nitric acid (4 µL, 1 mM), which was used for dissolution of the Cu^2+^ analyte. The calibration spectrum, ${\rm{\Gamma }}_1^{\left( {\rm{I}} \right)}\left( {{B_0}} \right)$, is shown in Fig. 3a (*green diamonds*). The strength of the external B-field was determined directly from the Zeeman splitting of the NV |±1〉 energy states. The magnetic field gradient over the FOV was <0.4% (Supplementary Fig. 1). The calibration spectrum was described well by a single Lorentzian fit. The peak at 511.3 ± 0.5 G corresponds to interactions between the NV probes and surface electronic spins with an average 〈*g*〉 = 2.01 ± 0.02. The half-width of the calibration spectrum *ω* = 60.4 ± 3 × 10^6^ rad s^−1^ indicates that the dominant contribution to the linewidth is a spin relaxation process on the time scale of tens of ns, consistent with surface spin phonon relaxation being the dominant contribution to the intrinsic spectrum^30^. A surface spin density of 2.4 spins per nm^2^ can be estimated from the intrinsic spectrum (Supplementary Note 6). Our method thus provides rapid and quantitative spectroscopy of the surface spin noise spectrum and can be used in combination with a variety of surface chemical passivation modes in order to understand and mitigate these effects.

With the intrinsic calibration spectrum ${\rm{\Gamma }}_1^{\left( {\rm{I}} \right)}\left( {{B_0}} \right)$ obtained, quantitative spectroscopy can be performed on the spin target by acquiring the combined *T*_1_ relaxation rate spectrum ${\rm{\Gamma }}_1^{\left( {{\rm{I}} + {\rm{Cu}}} \right)}\left( {{B_0}} \right)$. Figure 3a (*blue circles*) presents the combined spectrum from a 4 µL (100 mM) droplet of Cu^2+^ solution. The Cu^2+^ spectrum, ${\rm{\Gamma }}_1^{\left( {{\rm{Cu}}} \right)}\left( {{B_0}} \right)$ is then obtained by simply subtracting the calibration spectrum as shown in Fig. 3b. The Cu^2+^ spectrum is de-convolved in Supplementary Note 4 using the measured NV filter function; however, since the width of the filter function ~ 4 MHz (∆*ω* = 25.1 × 10^6^ rad s^−1^) is significantly less than the half linewidth of the Cu^2+^ spectrum (∆*ω* = 1.8 × 10^9^ rad s^−1^) the relaxation rate spectrum itself represents the spectral density of the hexaaqua-Cu^2+^ environment. For comparison, we have plotted the microwave absorption spectrum obtained from conventional continuous wave (CW)-EPR spectrometer. The plots in Fig. 3b were independently normalised to the area under the spectrum and show excellent agreement between the QPRM spectrum from the FOV (*red circles*) and the conventional CW-EPR spectrum. We emphasise here that the hexaaqua-Cu^2+^ spectral density is obtained by simply bringing the target spin into resonance, with no active driving of the target spin.

The Cu^2+^ spectrum can be derived theoretically and is given by the following expression (Supplementary Note 1):

3 $${\rm{\Gamma }}_1^{\left( {{\rm{Cu}}} \right)}\left( {{B_0}} \right) = \frac{{b_{{\rm{Cu}}}^2}}{2}\frac{{{\rm{\Gamma }}_2^{\left( {{\rm{I}} + {\rm{Cu}}} \right)} + {R_{{\rm{Cu}}}}}}{{{{\left( {{\rm{\Gamma }}_2^{\left( {{\rm{I}} + {\rm{Cu}}} \right)} + {R_{{\rm{Cu}}}}} \right)}^2} + {{\left( {2\pi D - \left( {{\gamma _{_{{\rm{NV}}}}} + {\gamma _{{\rm{Cu}}}}} \right){B_0}} \right)}^2}}},$$

where the *γ*_Cu_ is the gyromagnetic ratio of the Cu^2+^ spins, *b*_Cu_ characterises the strength of the overall probe–target coupling (Supplementary Note 1), and *R*_Cu_ is the total width of the spectral density arising from various processes intrinsic to the Cu^2+^ solution, e.g. intrinsic relaxation ($R_{{\rm{Cu}}}^{{\rm{Relax}}}$), dipole–dipole interactions ($R_{{\rm{Cu}}}^{{\rm{dip}}}$), spatial $( {R_{{\rm{Cu}}}^{{\rm{Spatial}}}} )$ and rotational motion ($R_{{\rm{Cu}}}^{{\rm{Rot}}}$). Our analysis shows that the dominant contribution to the linewidth arises from the intrinsic fluctuation rate, i.e. ${R_{{\rm{Cu}}}} \approx R_{{\rm{Cu}}}^{{\rm{Relax}}}$which is concentration independent and of order GHz (see Supplementary Note 2 for further details)^29^.

By fitting Eq. (3) to the Cu^2+^ spectrum, the depth of the NV probes, *h*_probe_, and the Cu^2+^ intrinsic fluctuation rate, $R_{{\rm{Cu}}}^{{\rm{Relax}}}$ can be obtained. The fit to the theoretical model, shown in Fig. 3b, is in excellent agreement with the data and yields an estimated probe depth of *h*_probe_ = 6.7 ± 0.04 nm and $R_{{\rm{Cu}}}^{{\rm{Relax}}}$ = (1.8 ± 0.05) × 10^9^ rad s^−1^. The NV depth is consistent with molecular dynamics simulations^31^. The Cu^2+^ intrinsic fluctuation rate, $R_{{\rm{Cu}}}^{{\rm{Relax}}}$ is in excellent agreement with the value obtained from bulk EPR of $R_{{\rm{Cu}}}^{{\rm{Relax}}}$ = (1.800 ± 0.005) × 10^9^ rad s^−1^, validating our experiment and theoretical approach. The peak position of the spectrum (*B*_res_ = 486 ± 1 G) is directly related to the *g*-factor of Cu^2+^ spins by $\left\langle {{g_{{\rm{Cu}}}}} \right\rangle = \frac{{hD}}{{{\mu _B}\,{B_{{\rm{res}}}}}} - {g_{NV}}$, where *h* is Planck’s constant, *D* is the zero field splitting of diamond, *μ*_B_ is the Bohr magneton, *g*_NV_ is the effective *g*-factor of the NV spin 2.0028^7^ and *B*_res_ is the magnetic field value corresponding to the peak of the spectrum. From the theoretical fit the measured value of $\left\langle {{g_{{\rm{Cu}}}}} \right\rangle$ = 2.21 ± 0.02 is in excellent agreement with the literature^32^.

The spectral resolution of our technique is ultimately limited by the point spread function (PSF) of the NV filter function. The width of the PSF is defined by hyperfine and free induction decay mechanisms (Γ_2_). For the NV array used in this work the PSF is ~ 4 MHz or 0.14 mT. Improvements such as isotopically pure ^12^C diamond would improve this resolution down to a few hundred kHz^9^. In the following section, we quantitatively investigate the spectroscopic electronic spin imaging via QPRM.

### High-resolution EPR imaging and spectroscopy

The Cu^2+^ spectrum shown in Fig. 3b was obtained by integrating the signal over the entire FOV at each applied magnetic field. To investigate the spectroscopic spatial resolution of the QPRM we performed a separate experiment, and interrogated a subset of pixels. The goal was to determine the *T*_1_ relaxation rates of the NV probes from a target sensing volume defined by the imaging pixel size and probe depth. The Cu^2+^ spectrum obtained from a region of interest (ROI) of 1.6 × 1.6 µm^2^ is shown in Fig. 3b (*green circles*) and is in excellent agreement with the spectrum obtained over the entire FOV. To investigate the uniformity of the measured signal across the imaging area we plot the measured *g*-factor from each ROI pixel as a histogram in Fig. 3c. The distribution is uniform over the imaging area with a mean of 〈*g*_Cu_〉 = 2.19 ± 0.03, consistent with the results for the FOV. A similar procedure was conducted for the calibration spectrum; this yielded a narrow distribution centred at 〈*g*_surface_〉 = 2.003 ± 0.007 consistent with the *g*-factor for free electrons (Fig. 3c).

At this point, we quantify the number of detected spins per sensing volume. The ROI sensing voxel is defined by 1.6 × 1.6 × 0.01 µm^3^ (0.025 µm^3^) or 25 aL (Supplementary Note 5). For 100 mM Cu^2+^ this equates to the detection of 1.5 × 10^6^ Cu^2+^ spins or ~ 2 amol per voxel. This is by no means the detection limit. To determine the sensitivity of the system the concentration of Cu^2+^ ions in solution was varied whilst on resonance with the NV probes, as shown in Fig. 3d. Using the NV probe height and intrinsic Cu^2+^ fluctuation rate, $R_{{\rm{Cu}}}^{{\rm{Relax}}}$ obtained from the Cu^2+^ spectrum (FOV) we can fit the concentration data using the following expression (Supplementary Note 1, Supplementary Eq. (11)):

4 $$\begin{array}{*{20}{l}}{\rm{C}}{{\rm{u}}^{2 + }} = \frac{{4.35 \times {{10}^{10}} \cdot {\rm{\Gamma }}_1^{\left( {{\rm{Cu}}} \right)}\left( {{B_{{\rm{res}}}}} \right) \cdot h_{{\rm{probe}}}^3{{\left( {{\rm{\Gamma }}_2^{\left( {{\rm{I}} + {\rm{Cu}}} \right)} + R_{{\rm{Cu}}}^{{\rm{Relax}}}} \right)}^2}}}{{\left( {\Gamma _2^{\left( {{\rm{I}} + {\rm{Cu}}} \right)} + R_{{\rm{Cu}}}^{{\rm{Relax}}}} \right)}}  {{\rm{mol}}\,{{\rm{l}}^{ - 1}}} , \hfill \end{array}$$

where ${\rm{\Gamma }}_1^{\left( {{\rm{Cu}}} \right)}\left( {{B_{{\rm{res}}}}} \right)$ is the decay rate from the Cu^2+^ spins measured on resonance, *h*_probe_ = 6.7 nm, $\Gamma _2^{\left( {{\rm{I}} + {\rm{Cu}}} \right)}$ = 4 MHz and $R_{{\rm{Cu}}}^{{\rm{Relax}}} = 1.8 \times {10^9}\,{\rm{rad}}\,{{\rm{s}}^{ - 1}}.$ The minimal detectable concentration was determined from the point at which the *T*_1_ measurement uncertainty was equivalent to the measured change in *T*_1_ in Fig. 3d. This corresponds to a minimum detectable number of spins for a single voxel of ~ 75,000 or ~ 125 zmol. To date, ambient EPR systems exhibit spin sensitivities of order 2 × 10^8^ G^−1^ Hz^−1/2^ ^33, 34^. Given our intrinsic linewidth of 4 MHz (1.4 G) our technique represents a 10^4^ improvement in spin sensitivity.

**Fig. 4:**
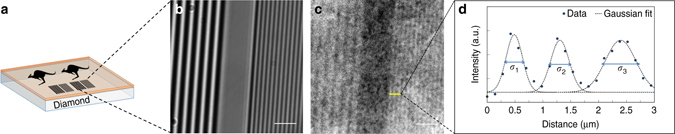
Spatial resolution of quantum probe relaxation microscopy. **a** Schematic diagram of the diamond with patterned PMMA layers. **b** Bright-field image of the grating mask used to determine the image resolution. **b** Single *τ*_sp_ image of Cu^2+^ ions at 460 G (*τ*_sp_ = 124 µs, *n* = 12 × 10^6^). The *light regions* indicate areas of Cu^2+^. *Scale bar* in **b**, **c** is 5 µm. **d** Line cut through a section of the quantum probe relaxation image (*yellow line*). The Gaussian fit to each peak in the line scan shows linewidths of *σ*_1_ = 290 ± 30, *σ*_2_ = 330 ± 40 and *σ*_3_ = 540 ± 60 nm. The minimum linewidth is consistent with the diffraction limit of the microscope, ∼305 nm

To quantify the ultimate spatial imaging resolution, we move to a separate region of the imaging chip with an image mask comprised of a series of gratings of width ~ 500 nm and pitch of 1 µm as shown in Fig. 4a. The QPR image at 460 G shown in Fig. 4b reveals the Cu^2+^ spatial distribution. A line cut through a series of grating lines is shown in Fig. 4c, demonstrating an imaging resolution of 290 ± 30 nm which is in excellent agreement with the optical resolution of the microscope 1.22*λ*/(2NA) ≈ 305 nm.

These results show that spatial imaging of Cu^2+^ ions with diffraction limited resolution and zeptomol sensitivity can be achieved under ambient conditions.

### Monitoring redox reactions kinetics

**Fig. 5:**
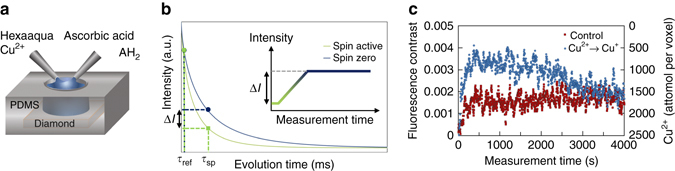
Monitoring redox kinetics. **a** Schematic showing the redox reaction of Cu^2+^ ions (spin ½) to cuprous ions (spin zero) via ascorbic acid at a ratio of 1:1. **b** Dynamic quantum probe relaxation microscopy using a two point *T*_1_ measurement scheme to detect redox reactions of spin active molecules. The first time point, *τ*_ref_, is used to normalise the fluorescence signal over the entire FOV (50 × 50 µm) with the second time point *τ*_sp_ set to the *T*_1_ time of the NV probes. By monitoring the normalised intensity over time, changes in the *T*_1_ rate will manifest as a change in the fluorescence intensity as shown schematically in the inset of **b**. **c** Two-point *T*_1_ measurement showing the control experiment (*red squares*) consisting of a dilution of the Cu^2+^ from 100 mM to 50 mM with water and the reduction of Cu^2+^ using ascorbic acid (*blue circles*). The Cu^+^ is found to disproportionate/re-oxidise over a period of 1 h. The fluorescence intensity was determined from the FOV at measurement intervals of 3 s

Finally, we demonstrate dynamic spin detection by monitoring the redox state of Cu^2+^ ions in the presence of a reducing agent, ascorbic acid (AH_2_) as described in Fig. 5a. To capture dynamic redox changes, we implement a two-point *T*_1_ measurement scheme with an external magnetic field set to Cu^2+^ ↔ NV resonant point *B*_0_ = 486 G (Fig. 5b). The reaction kinetics for Cu^2+^ in the presence of ascorbic acid can be described by an anaerobic chain-beginning reaction 2Cu^2+^ + AH_2_ ↔ 2Cu^+^ + A + 2H^+^. The low value of the equilibrium constant, *K*_e_ = (5 ± 2) × 10^−9^ M^2^ ^35^, suggests that after the initial reduction to Cu^+^ the Cu^+^ re-oxidises back to Cu^2+^ which dominates at long times, consistent with the expected disproportionation of Cu^+^ ions.

Figure 5c (*red squares*) shows a control measurement performed by diluting the Cu^2+^ 100 mM solution by 1:1 with MilliQ water. The integrated fluorescence intensity from the FOV was recorded at 3 s intervals over 4000 s. The *t* = 0 point represents the time MilliQ water was added. The fluorescence intensity change (0.2%) is consistent with the change in *T*_1_ decay expected when the Cu^2+^ concentration is reduced from 100 to 50 mM. The short time oscillations observed in the control curves arise from fluctuations in the laser excitation intensity. These fluctuations do not impact the long-term reduction and oxidation trends in the data and can be reduced using active feedback on the laser modulation. The measured fluorescence intensity is proportional to the Cu^2+^-induced decay rate and is given by Eq. (5), see Supplementary Note 7 for details:

5 $${\rm{\Gamma }}_1^{\left( {{\rm{Cu}}} \right)}\left( {{B_{{\rm{res}}}}} \right) = \frac{{\Delta I}}{{c\,{\tau _{{\rm{sp}}}}\Delta {I_0}}},$$

where *τ*_sp_ is the measurement time point in the two-point *T*_1_ measurement sequence, $\frac{{\Delta I}}{{\Delta {I_0}}}$ is the percentage change in fluorescence intensity and *c* = 0.04 is the spin relaxation contrast governed by the NV ensemble.

Equations (4) and (5) can be used to translate the change in fluorescence intensity into the Cu^2+^ concentration. This allows the Cu^2+^ concentration per voxel to be determined over time as shown in Fig. 5c. To investigate dynamic redox reactions, we introduce ascorbic acid (AH_2_) at a ratio of 1:1 with Cu^2+^ ions. Figure 5c (*blue squares*) shows the two point *T*_1_ measurement from the FOV over the same time period as the control.

The ascorbic acid was found to reduce ~ 60% of the Cu^2+^ ions in the FOV within 500 s. The reduced spin-zero cuprous ions are not stable in solution and slowly convert back into Cu^2+^ via disproportionation and/or aerobic oxidation after 4000 s. The final intensity returns to that of the diluted control concentration. This demonstration shows that the redox kinetics from sensing volumes of ~ 25 fL can be monitored over time and with a sensitivity of order attomol.

## Discussion

In summary, we have demonstrated a QPRM using an array of NV probes in diamond. The microscope operates at room temperature and in ambient conditions and allows simple non-invasive spectroscopy of unpaired electron spin systems in aqueous solution and under biologically compatible conditions. In demonstrating the system, we have focused on the spectroscopy and imaging of the hexaaqua-Cu^2+^ complex in aqueous solution. We have demonstrated species-specific spatial imaging of the spin target with diffraction limited resolution at 300 nm and ultimate spin sensitivity approaching the zeptomol level. We have shown how quantitative spectroscopic imaging can be performed on external Cu^2+^ spins in sensing volumes down to 25 aL with micrometre spatial resolution. The theoretical framework describing the interaction of the target spin system and the NV spin probe is in excellent agreement with the experimental data.

At present, the transition metal detection levels in these initial demonstrations are several orders of magnitude higher than those found in typical biological environments. However, straightforward improvements in material design of the diamond imaging chip will dramatically reduce the acquisition time and improve the sensitivity towards unpaired electron spins. For example improving the conversion efficiency of NV centres in our imaging chip from 1 to 10%^36^ combined with photon collection efficiency gains^37^ would see a factor of 20 improvement in the fluorescence signal which would allow sub-second acquisition times and redox kinetics from just tens of zeptomoles of analyte. Furthermore, improved surface passivation of the diamond to remove unwanted surface electron spins will bring an additional order of magnitude improvement in spin sensitivity^38^.

The application of quantum control to the detection and imaging of electronic spin systems represents a significant step forward. The work reported here demonstrates that quantum sensing systems can accommodate the fluctuating Brownian environment encountered in ʽreal’ chemical systems and the inherent fluctuations in the spin environment of ions undergoing ligand rearrangement. A key feature of the technique is the high sensitivity achieved by virtue of the high axial resolution. There are a variety of approaches which can be used to bring systems of interest into close proximity with the diamond imaging chip including chemical functionalisation routes which are now well established for diamond^39^ or, in the case of cell membrane systems, targets may be formed or adhered directly on the surface of the diamond^15^. Therefore, QPRM represents a promising approach for probing fundamental nanoscale biochemistry such as binding events^40^ catalytic reactions, electron transfer processes and the intra-cellular transition metal concentration in the periplasm of prokaryotic cells^41^.

## Methods

### Materials

The diamond imaging sensor used in this work is engineered from electronic grade Type IIa diamond (Element 6). The diamonds were thinned, cut and re-polished to a 2 × 2 × 0.1 mm^3^ crystal (DDK, USA). NV defects were engineered via ion implantation of ^15^N atoms at an energy of 4 keV and dose of 1 × 10^13^ ions per cm^2^. Molecular dynamic simulations indicate a NV depth range between 5 and 10 nm^31^ which is consistent with the depth measurement from our analysis of 6.7 nm. The implanted sample was annealed at 1000 °C for 3 h and acid treated to remove any unwanted surface contamination. The density of NV centres post annealing was 1 × 10^11^ NV per cm^2^. The linewidth of the ODMR was typically 4 MHz. The hyperfine spectrum from the ^15^N implant could not be resolved due to the inhomogeneous broadening from the dipole coupling of ^13^C spins present in the material at a concentration of 1.1%. The typical *T*_1_ time of the array was 1.8 ms off resonance with a de-phasing time *T*_2_ of ~ 2 µs.

### Optical imaging

The wide-field imaging was performed on a modified Nikon inverted microscope (Ti-U). Optical excitation from a 532 nm Verdi laser was focused (*f* = 300 mm) onto an acousto-optic modulator (Crystal Technologies Model 3520-220) and then expanded and collimated (Thorlabs beam expander GBE05-A) to a beam diameter of 10 mm. The collimated beam was focused using a wide-field lens (*f* = 300 mm) to the back aperture of the Nikon ×60 (1.4 NA) oil immersion objective via a Semrock dichroic mirror (Di02-R561-25 × 36). The NV fluorescence was filtered using two bandpass filters before being imaged using a tube lens (*f* = 300 mm) onto a sCMOS camera (Neo, Andor). Microwave excitation to drive the NV spin probes was applied via an omega gold resonator (diameter = 0.8 mm) lithographically patterned onto a glass coverslip directly under the diamond imaging chip. The microwave signal from an Agilent microwave generator (N5182A) was switched using a Miniciruits RF switch (ZASWA-2-50DR+). The microwaves were amplified (Amplifier Research 20S1G4) before being sent to the microwave resonator. A Spincore Pulseblaster (ESR-PRO 500 MHz) was used to control the timing sequences of the excitation laser, microwaves and sCMOS camera and the images where obtained and analysed using custom LabVIEW code. The excitation power density used for imaging was 30 W per mm^2^ and all images were taken in an ambient environment at room temperature.

### Image mask

The fabrication of the image mask was done by cleaning the diamond imaging chip in hot acetone (65 °C) followed by rinsing in isopropanol alcohol (IPA) and deionised water. The sample was then spin-coated with 280 nm thick PMMA A4 950k resist. After the spin-coating the sample was baked on the hot plate for 15 min at 100 °C followed by additional 10 min at 170 °C. This enables a better solvent evaporation and prevents the resist from outgassing in vacuum. A 30 nm thick conduction layer of Cr was deposited on the resist at the rate of 0.2 A s^−1^. The conduction layer provides charge dissipation during the electron beam lithography (EBL) exposure. The sample was exposed to create a desired pattern using a 100 keV EBPG5000+EBL system. The exposed resist was then developed in a mixture of methyl isobutyl ketone (MIBK) and IPA in the ratio of 1:3. The development was performed for 1 min and the sample was then rinsed in a fresh IPA and deionised water.

### Image analysis

Image analysis was performed using custom LabVIEW code. The *T*_1_ relaxation images were obtained by determining the *T*_1_ decay curve at each pixel and fitting the data to a stretched exponential of the form $y = A\,{\rm{exp}}\,\left( {{{\left( {t{\rm{/}}{T_1}} \right)}^p}} \right) + c$, where *A* is the amplitude of the exponential decay, *T*_1_ is the spin lattice relaxation time, *p* is the stretched exponential power (*p* = 1 represents a single exponential decay) and *c* is the offset. Near surface NV defects are known to exhibit a distribution of *T*_1_ times from the NV ensemble depending on their proximity to the surface and local spin environment^18^. This distribution leads to a non-exponential *T*_1_ decay which is characterised well by a stretched exponential function. The amplitude and offset are left as free parameters since these can vary depending on the alignment of the magnetic field particularly around the excited-state level anti-crossing (ESLAC) at 512 G.

### Magnetic field alignment

Magnetic field alignment was achieved by monitoring the NV fluorescence signal near the ESLAC as described in ref. ^42^. The fluorescence at 512 G was extremely sensitive to the field alignment and could be aligned with a particular axis to a precision better than 0.1°. The magnetic field gradient is characterised across the FOV by determining the Zeeman splitting at each pixel via ODMR (Supplementary Note 3). The measured field gradient in the *y* direction was 0.4% and 0.07% in the *x* direction.

### Continuous wave EPR

Continuous-wave EPR spectra were acquired using a CMS8400 X-band (9.4 GHz) spectrometer (Adani, Belarus) fitted with a TE102 cavity and operating at a fixed receiver time constant of 100 ms and 100 kHz magnetic field modulation. Solution measurements were made at room temperature using a quartz flat cell (Wilmad, WG-808-Q).

### Data availability

The data that support the findings of this study are available from the corresponding author upon reasonable request.

## Electronic supplementary material

Supplementary Information
