# Supplementary material for: Electron paramagnetic resonance microscopy using spins in diamond under ambient conditions
Source: Nat Commun. 2017 Sep 6;8:458. doi: 10.1038/s41467-017-00466-y (PMC5587709; doi:10.1038/s41467-017-00466-y)
Supplement: Supplementary file 1 — Supplementary Information [file 41467_2017_466_MOESM1_ESM.pdf]

Description of Supplementary Files

File Name: Supplementary Information
Description: Supplementary Figures, Supplementary Notes and Supplementary References

Supplementary Note 1: Determination of T_1 relaxation rate spectrum

The population decay of the $|0\rangle$ state of an NV spin in the presence of an environmental transverse magnetic field of magnitude, *b* and frequency, ω_{E}, is described by:

$$P(t) = \frac{1}{2} + \frac{1}{2}\exp^{-\frac{b^2\Gamma_2}{2(\Gamma_2^2+\delta^2)}} \ , \tag{1}$$

where δ is the detuning between the NV energy levels, $\omega_{\mathrm{NV}} = 2\pi D - \frac{g_{\mathrm{NV}}\mu_{\mathrm{B}}}{\hbar}B_0 \equiv 2\pi D - \gamma_{\mathrm{NV}}B_0$ and the frequency of the environment, ω_{E}; B_0 is the strength of the axial magnetic field; $D = 2.87\ GHz$ is the crystal field splitting of diamond, $g_{\mathrm{NV}} = 2.0028$ [1] is the effective g factor of the NV spin and $\Gamma_2$is the transverse relaxation rate of the NV centre.

$$\delta = 2\pi D - \frac{g_{\mathrm{NV}}\mu_{\mathrm{B}}}{\hbar}B_0 - \omega_{\mathrm{E}}. \tag{2}$$

An arbitrary magnetic environment will exhibit a distribution of frequencies defined by its frequency spectrum, $S^{(\mathrm{E})}(\omega_{\mathrm{E}}, B_0)$, which is a probability distribution for ω_{E}. Hence the NV population is given by the average of Supplementary Eq. 1 over this distribution. Because the distance of the NV to the interface is much larger than the average separation between environmental spins in solution, the short-time expansion of Supplementary Eq. 1 is still rather long compared to the timescales associated with the intrinsic dynamics of the environment. We measure T_1 times of µs to ms, yet environmental processes are of order GHz. The average relaxation rate is therefore approximately given by the average of the relaxation rate in Supplementary Eq. 1 over the environmental spectrum (Supplementary Eq. 3).

$$\frac{1}{T_1^{(\mathrm{E})}}(B_0) = \Gamma_1^{(\mathrm{E})}(B_0) \approx \int_{-\infty}^{\infty} \frac{b^2\Gamma_2}{2(\Gamma_2^2+\delta^2)} S^{(\mathrm{E})}(\omega_{\mathrm{E}}, B_0)d\omega_{\mathrm{E}}\ . \tag{3}$$

The measured relaxation rate from the NV centre is therefore a convolution of the NV filter function $G = \frac{b^2\Gamma_2}{2(\Gamma_2^2+\delta^2)}$ and the spectral density of the environment. It is important to note the tuning of the NV filter function can be achieved by application of an external magnetic field which Zeeman split the |-1⟩ and |+1⟩ energy levels modifying the NV probe frequency. Denoting the filter function as G, we arrive at the general expression for the measured NV relaxation rate spectrum in the presence of an arbitrary magnetic environment.

$$\frac{1}{T_1^{(\mathrm{E})}}(B_0) = \Gamma_1^{(\mathrm{E})}(B_0) = \int_{-\infty}^{\infty} G(\Gamma_2, \omega_{\mathrm{E}}, B_0)\, S^{(\mathrm{E})}(\omega_{\mathrm{E}}, B_0)d\omega_{\mathrm{E}}. \tag{4}$$

For the particular case of Cu^{2+} ions studied in this work, we note that the state of the Cu^{2+} environment is self-correlated in an oscillatory fashion at its Larmor frequency, $\omega_{\mathrm{E}} = \frac{g_{\mathrm{Cu}}\mu_B}{\hbar}B_0 \equiv \gamma_{Cu}B_0$ because, regardless of their initial state, environmental spins will be pointing the other way after time, $t = {}^{\pi}/_{\omega_0}$ and will return to their initial orientation after time, $t = {}^{2\pi}/_{\omega_0}$. These correlations will, however, decay on the timescale over which environmental processes (such as spin-flips, rotational diffusion, spatial diffusion in and out

of the sensing volume, etc.) either re-orient or substitute these spins. Thus, the autocorrelation function of the environmental field is given by:

$$\langle B(t)B(0)\rangle = \exp(-R_{\mathrm{Cu}}t)\cos(\omega_0 t)\,, \tag{5}$$

where $\mathrm{R}_{\mathrm{Cu}} = R_{\mathrm{Cu}}^{\mathrm{dip}} + R_{\mathrm{Cu}}^{\mathrm{Spatial}} + R_{\mathrm{Cu}}^{\mathrm{Rot}} + R_{\mathrm{Cu}}^{\mathrm{Relax}}$ is the fluctuation rate of the Cu^{2+} spins, due to spin-spin interactions, spatial diffusion, rotational diffusion, and vibrational relaxation, respectively.

To get the Cu^{2+} spectrum required for Supplementary Eq. 3, we simply take the Fourier transform (appropriately normalised since $S^{(\mathrm{Cu})}(\omega_{\mathrm{Cu}})$ is a probability distribution) of Supplementary Eq.4 to give a spectral distribution of

$$S^{(\mathrm{Cu})}(\omega_{\mathrm{Cu}}, B_0) = \frac{2}{\pi}\frac{R_{\mathrm{Cu}}}{R_{\mathrm{Cu}}^2+(\omega_{\mathrm{Cu}}-\omega_0)^2}\,. \tag{6}$$

Using Supplementary Eqs. (3) and (5) we find

$$\Gamma_1^{(\mathrm{Cu})}(B_0) = \frac{b_{\mathrm{Cu}}^2}{2}\frac{\Gamma_2+R_{\mathrm{Cu}}}{(\Gamma_2+R_{\mathrm{Cu}})^2+[2\pi D_{ZFS}-(\gamma_{\mathrm{NV}}+\gamma_{\mathrm{Cu}})B_0]^2} \tag{7}$$

Now all that's left to be determined is the environmental field strength b and the environmental relaxation rate/spectral width.

The derivation above describes the measured relaxation rate of the NV in the presence of a Cu^{2+} spin environment. In practise the NV spin probes are also exposed to their intrinsic spin bath consisting of electronic and nuclear spins in the bulk diamond and on the surface. Therefore the relaxation rate spectrum of the NV needs to be characterised and accounted for before introducing the target system of interest. This is done by performing a calibration spectrum is the absence of the target system and then repeating the spectrum with the target. Since the relaxation rate spectrum is dictated by the addition of these spectral components, the subtraction of the calibration measurement is all that is required to recover the target spectrum alone.

Effective environmental field strength/coupling of Cu^{2+} to NV

The effective field strength of the Cu^{2+} is related to the components of the NV-Cu^{2+} magnetic dipole tensor via

$$b_{\mathrm{single}} = \frac{1}{2}\left[\left(B_{\mathrm{xx}} + B_{\mathrm{yy}}\right)^2 + 4B_{\mathrm{xy}}^2\right]^{1/2},$$

$$= \frac{3}{2}\frac{b}{R^3}\sin^2\theta\,, \tag{8}$$

where $b = \frac{\mu_0}{4\pi\hbar} g_{\mathrm{NV}} g_{\mathrm{Cu}} \mu_B^2$, θ is the angle between their separation vector and the direction of the external field, and R is their separation distance. To determine the total effect of *n*

spins in the environment, we assume the environmental spins constitute a continuum (justified by the fact that Cu^{2+}-Cu^{2+} separations are much smaller than NV-Cu^{2+} separations), and integrate Supplementary Eq. 1 over the environment. That is, with $R = \sqrt{x^2 + y^2 + z^2}$ and $\sin^2\theta = 1 - \left(\frac{z}{r}\right)$

$$b_{\text{tot}}^2 = \frac{9}{4}\int_{h_{\text{probe}}}^{\infty}\int_{-\infty}^{\infty}\int_{-\infty}^{\infty}\frac{n}{R^6}\left(1 - \frac{z^2}{R^2}\right)^2 dxdydz\,,$$

$$= \frac{\pi}{8}\frac{b^2 n}{h_{\text{probe}}^3}\,,$$

$$= 10^3 \times \frac{\pi}{8}\frac{b^2 c N_A}{h_{\text{probe}}^3}\,,$$

$$= 4.6 \times 10^{-11}\left(\frac{c}{h_{\text{probe}}^3}\right)\ \text{rad}^2\text{s}^{-2}\text{m}^3\text{M}^{-1} \qquad (9)$$

where c is the concentration in mol/L, and h_{probe} is the depth of the NV array below the diamond-electrolyte interface.

For the case of a concentration of 0.1 M and a NV depth of 6.7 nm (determined from fit to relaxation rate spectrum in manuscript), we find

$$B_{\text{tot}} = 3.9 \times 10^6\ \text{rad s}^{-1} = 620\ \text{kHz} = 22.1\ \mu\text{T} = 0.22\ \text{G}. \qquad (10)$$

Using the concentration dependence of b_{tot}^2 from Supplementary Eq. (9) we can re-write Supplementary Eq. (7) in terms of the dependence on Cu^{2+} concentration.

$$\text{Cu}^{2+}\ [\text{mol/L}] = \frac{4.35\times10^{10}\cdot\Gamma_1^{(\text{Cu})}(B_{\text{res}})\cdot h_{\text{probe}}^3\left(\Gamma_2^{(\text{I+Cu})}+R_{\text{Cu}}^{\text{Relax}}\right)^2}{\left(\Gamma_2^{(\text{I+Cu})}+R_{\text{Cu}}^{\text{Relax}}\right)} \qquad (11)$$

Supplementary Note 2: NV relaxation mechanisms

Dipole-dipole mediated relaxation of Cu^{2+} spins

The dipole-dipole coupling strength between two Cu^{2+} spins is given by:

$$R_{\text{Cu}}^{\text{dip}} = \frac{\mu_0}{4\pi}\hbar\left(\frac{g_{\text{Cu}}\mu_B}{\hbar}\right)^2\frac{1}{r^3}\,. \qquad (12)$$

From previous work, the distribution of distances from a spin to it's nearest neighbour is given by [2]:

$$P_r(r) = 4\pi n r^2 \exp\left[-\frac{4}{3}\pi n r^3\right],$$

which gives an average separation of

$$\langle r\rangle = \int_0^\infty r\,P_r(r)dr\,, \qquad (13)$$

$$= 0.55\, n^{-1/3}.$$

Substituting this into Supplementary Eq. 8 gives:

$$R_{\mathrm{Cu}}^{\mathrm{dip}} = 1.71 \times 10^{9}\ \mathrm{rad\ s^{-1}M^{-1}} \times c = 272\ \mathrm{MHz\ M^{-1}} \times c\ . \qquad (14)$$

For a concentration of c = 0.1 M we have, $R_{\mathrm{Cu}}^{\mathrm{dip}} = 1.71 \times 10^{8}\ \mathrm{rad\ s^{-1}} = 27\ \mathrm{MHz} = 9.7\ \mathrm{G}$, which is smaller than the observed linewidth.

Spectral diffusion of Cu^{2+} spins

The spatial diffusion rate of molecules in water has been derived in previous work [3].

$$R_{\mathrm{Cu}}^{\mathrm{Spatial}} = D_{\mathrm{Cu}} \left(\frac{3}{4h_{\mathrm{probe}}}\right)^{2}, \qquad (15)$$

where D_{Cu} is the self-diffusion rate of Cu^{2+} spins (not to be confused with the zero-field splitting of the NV). Noting that the self-diffusion rate for water at standard temperature and pressure is $3 \times 10^{-9}\ \mathrm{m^2s^{-1}}$ and that Cu^{2+} is larger and heavier and will therefore have a much slower diffusion rate than water and again taking an NV depth of 6.7 nm, $R_{\mathrm{Cu}}^{\mathrm{Spatial}} = 37 \times 10^{6}\ \mathrm{rad\ s^{-1}} = 2.1\ \mathrm{G}$.

Taking into account all of the possible broadening mechanisms of Cu^{2+} on the NV it is clear that the dominant contribution to the spectral linewidth arises from the intrinsic relaxation rate of the Cu^{2+} spins.

Intrinsic relaxation and/or rotational diffusion

Both of these processes produce a Cu^{2+} fluctuation rate that is independent of both Cu^{2+} concentration and NV depth, and are therefore indistinguishable without additional information. The theoretical linewidth of due to the intrinsic relaxation of hexaaqua Cu^{2+} has been described in the literature and given by [4]:

$$\frac{1}{T_{1\mathrm{Cu}}} = 2.04 \times 10^{-4} \left(\left(\frac{T}{\eta}\right) + 0.23T^{2}\right) \qquad (16)$$

where T is the temperature and η is the viscosity of water $8.9 \times 10^{-4}\ \mathrm{Pa.s}$. Solving this equation for T= 22 °C gives a $R_{\mathrm{Cu}}^{\mathrm{Relax}}$ fluctuation rate of $2.5 \times 10^{9}\ \mathrm{rad\ s^{-1}}$.

This rate alone accounts for the width of the measured spectra suggesting that the contributions from spatial and rotational diffusion are negligible. To more accurately compare and quantify the linewidth of the spectrum obtained via quantum probe relaxation

microscopy we compare our spectrum to that obtained with traditional CW EPR in Figure 3b on the manuscript. Our linewidth and normalised amplitudes are in excellent agreement with one another and demonstrate a $R_{\text{Cu}}^{\text{Relax}}$ dominated width of $1.8 \times 10^{9}\ \text{rad s}^{-1}$

Supplementary Note 3: Magnetic field gradient

Quantum probe relaxation microscopy relies on the ability to bring a target spins into resonance with the NV spin probes. The external magnetic field in this work was applied via a permanent rare earth magnet (Dia=25mm) aligned at 35.3° from the normal of the <100> diamond surface. This external field direction addresses a particular orientation class of NV centres. Optimisation of the magnetic field alignment is achieved by monitoring the photo-luminescence (PL) from the imaging array at the excited state level anti-crossing (ESLAC) at ~512 G [6]. With the magnetic field aligned to the NV axis we perform an optically detected magnetic resonance (ODMR) scan over the field of view (FOV) to determine the Zeeman splitting from the aligned NV centres at each pixel. The Zeeman splitting can be converted to the external magnetic field using the NV gyromagnetic ratio of 2.8MHz/G. The resulting external magnetic field map is shown in the Supplementary Figure 1:

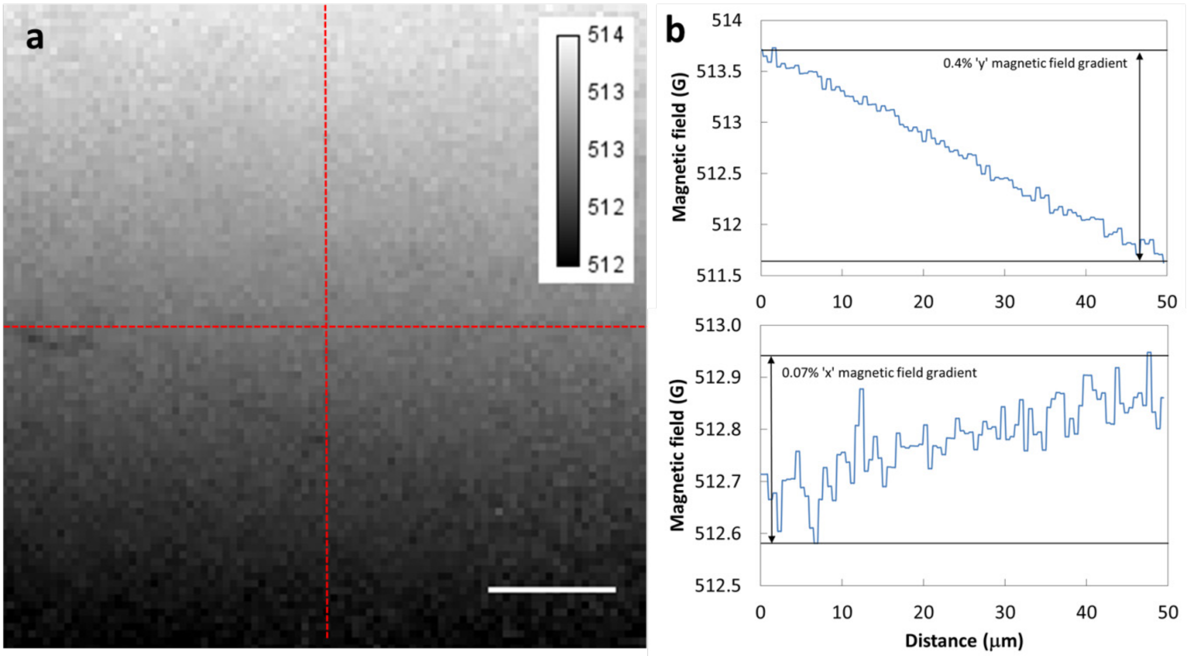

Supplementary Figure 1: a, Magnetic field map from a 50x50 μm imaging area. The calibration bar presents the strength of the magnetic field in gauss. The scale bar represents 10 μm. **b,** Line cuts in the x-dir (bottom) and y-dir (top) direction showing the strength of the B-field as a function of distance The maximum magnetic field gradient over the 50x50 μm FOV was 0.4%.

The magnetic field gradient observed here has a negligible impact of the determination of the relaxation rate spectrum from the Cu^{2+} spin targets. However, target molecules with a narrow spectral distribution require careful control and calibration of the external field. The ODMR field map can report the magnetic field strength spatially over the entire field of view

so the relaxation maps can be reconstructed on a pixel by pixel basis by performing an ODMR map at each magnetic field. Alternatively, basis magnets can be applied in the opposite direction to even out magnetic inhomogeneities [7] or we can move to [111] diamond where external magnetic field can be applied uniformly using an electromagnet or large permanent magnet [8].

Supplementary Note 4: De-convolution of the relaxation rate spectrum to obtain the spectral density

The measured relaxation rate spectrum obtained and presented in the manuscript can be de-convolved with the NV filter function to obtain the actual spectral density of the Cu^{2+} environment. As discussed above the width of the spectral density for Cu^{2+} ions is $1.8 \times 10^9\,\mathrm{rad\,s^{-1}}$. Considering the narrow linewidth of the NV filter function $\Delta\omega = 25 \times 10^6\,\mathrm{rad\,s^{-1}}$ (angular frequency) its effect on the measured linewidth is minimal. For completeness, Supplementary Figure 2 shows the de-convolved spectrum for Cu^{2+} with very little difference between the measured and de-convolved spectra. The de-convolution will be more valuable when characterising spin targets with much narrower spectral densities (see for example our previous work on the probing P1 spins intrinsic to the diamond [5]). For broad spin targets such as Cu^{2+} the relaxation rate accurately reproduces the spectral density.

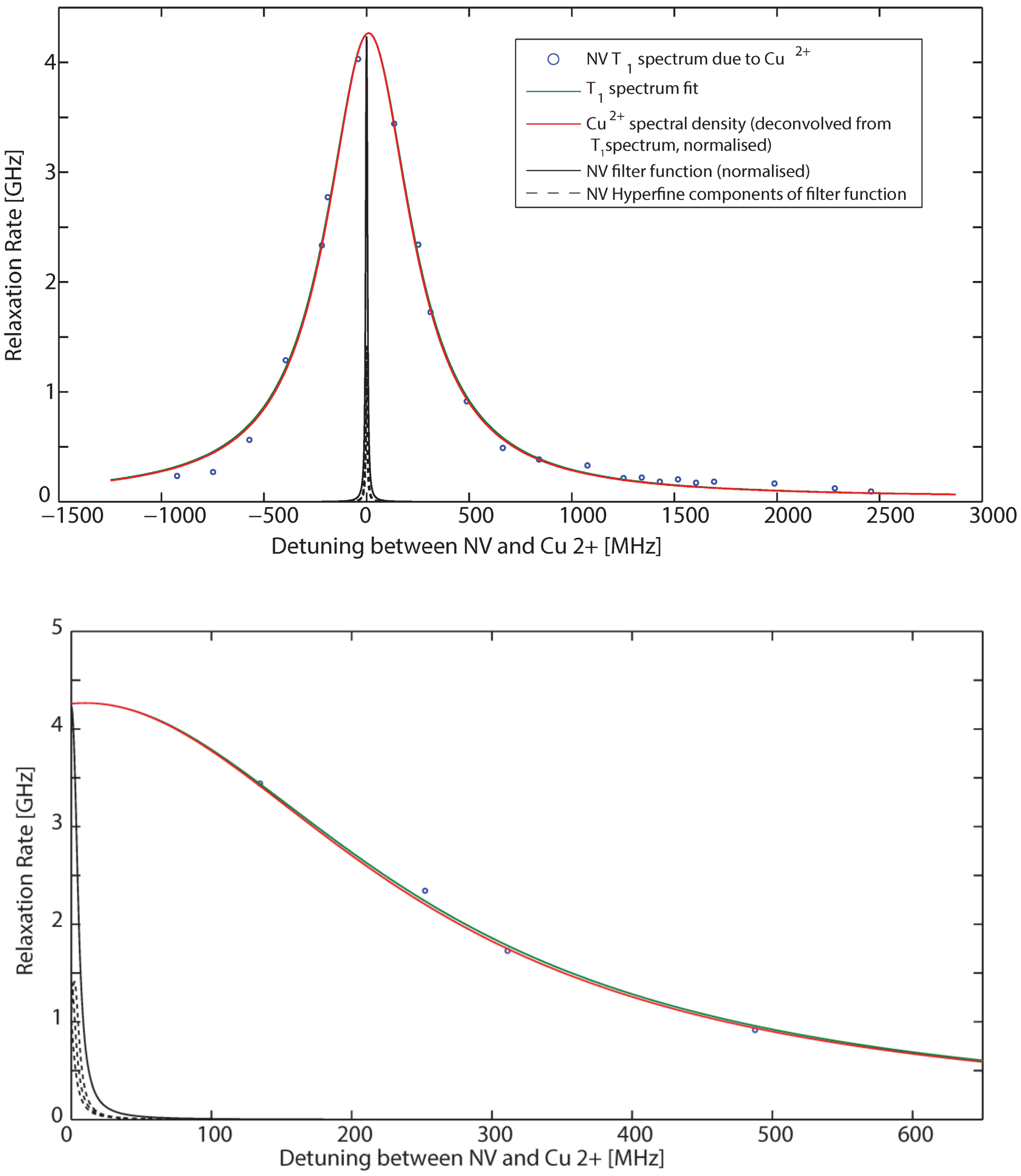

Supplementary Figure 2: De-convolved relaxation rate spectrum. Top, shows the measured relaxation spectrum in green and the de-convolved spectrum using the NV filter function of width 4 MHz. bottom: Zoomed in region of the spectrum, showing the minor reduction in linewidth after the de-convolution.

Supplementary Note 5: Depth dependence of the T_1 relaxation rate

If we consider a single spin placed at directly above the NV at height h, its variance is given by [3]:

$$\langle {B_{0,z}}^2 \rangle = \frac{4}{3} S(S+1) \frac{A^2}{h_{\text{probe}}^6} \quad , \tag{17}$$

where $A = \frac{\mu_0}{4\pi} \gamma_{\text{NV}} g_{\text{e}}$, h_{probe} is the depth of the probe beneath the diamond surface and S is the spin quantum number of the target.

The effective number of spins due to the entire environment is then:

$$N_{\text{eff}} = \frac{\langle {B_z}^2 \rangle}{\langle {B_{0,z}}^2 \rangle} = \frac{n\pi h_{\text{probe}}^3}{8} \quad , \tag{18}$$

with corresponding effective volume

$$V_{\text{eff}} = \frac{\pi h_{\text{probe}}^3}{8} \quad . \tag{19}$$

This quantity is a measure of the effective sensing volume of environmental spins to which the NV is exposed, which implies the effective spatial resolution of this sensing protocol is $\Delta L \sim 3/4\, h_{\text{probe}}$, which is ultimately limited by how closely an NV centre may reside to the diamond surface.

Supplementary Note 6: Estimate of surface spin density:

The rms field due to spins on the surface of the diamond may be determined by the approach described in [3]:

$$b_{\text{ss}}^{\text{rms}} = 277\ \mu\text{T}\ \text{nm}^{-3} \frac{\sigma^{1/2}}{h_{\text{probe}}^2} \quad , \tag{20}$$

where σ is the effective surface spin density and h_{probe} is the depth of the NV below the diamond surface.

From the surface spin relaxation spectrum, $\Gamma_1^{(\text{I})}(B_0)$, shown in Figure 3b of the manuscript the effective field strength from the surface spins can be determined with the knowledge of the amplitude and width of the spectrum. The variance of the field is given by:

$$\langle {b_{\text{ss}}^{\text{rms}}}^2 \rangle = 2\ \times \Gamma_1^{(\text{I})}(B_{\text{res}}) \times \Delta\omega \ , \tag{21}$$

$$\langle {b_{\text{ss}}^{\text{rms}}}^2 \rangle = 2\ \times 58095 \times 60.4 \times 10^6 = 7.01 \times 10^{12}\ \text{rad}^2\ \text{s}^{-2},$$

$b_{\rm ss}^{\rm rms} = \sqrt{7.01 \times 10^{12}} = 2.649 \times 10^{6}\ \text{rad s}^{-1} = 421.6\ \text{kHz} = \frac{421.6\ \text{kHz}}{2.8\ \text{MHz/G}} = 0.15\ \text{G} = 15\ \mu\text{T}\ .$

The field from the surface spins is related to the effective spin density via the following expression, see [3] for derivation.

$$b_{\rm ss}^{\rm rms} = 277\ \mu\text{T nm}^{-3} \frac{\sigma^{1/2}}{h_{\rm probe}^{2}}\ . \tag{22}$$

This assumes that the surface distribution effectively becomes planar arrangement. Thus, fixing $z = h$, and integrating over x and y, to obtain:

$$\sigma = \sqrt{\frac{b_{\rm ss}\, h_{\rm probe}^{2}}{277\ \mu\text{T nm}^{-3}}} = \sqrt{\frac{15\times 6.7^{2}}{277\ \mu\text{T nm}^{-3}}} = 2.4\ \text{spins/nm}^{2}. \tag{23}$$

Supplementary Note 7: Converting photo-luminescence into environmental decay rate

This final section describes how we can convert the measured photo-luminescence from the NV imaging array into an environmental decay rate. The time-dependent population of the NV $|0\rangle$ state in the absence of any external environment is given by:

$$P_0(t) = \exp\left(-\Gamma_1^{(\rm I)} t\right)\ . \tag{24}$$

The addition of some external environment (T) introduces an additional component to this decay, $\Gamma_1^{(\rm E)}$, as given by:

$$P(t) = \exp\left(-\left(\Gamma_1^{(\rm I)} + \Gamma_1^{(\rm T)}\right)t\right)\ . \tag{25}$$

The time between laser pulses, for which the system is allowed to evolve, is given by τ_m. Therefore the PL intensity will be proportional to these populations,

$$PL_0 = kP_0(\tau_{\rm sp})\ ,$$
$$PL = kP(\tau_{\rm sp})\ . \tag{26}$$

In terms of the external component of the relaxation rate, the change in PL is given by:

$$\Delta_{PL} = (PL_0 - PL) \times c\ ,$$
$$= k\left[P_0(\tau_{\rm sp}) - P(\tau_{\rm sp})\right] \times c\ ,$$
$$= k\ \exp\left(-\Gamma_1^{(\rm I)}\tau_{\rm sp}\right)\left[1 - \exp\left(-\Gamma_1^{(\rm T)}\tau_{\rm sp}\right)\right] \times c\ ,$$
$$= PL_0\left[1 - \exp\left(-\Gamma_1^{(\rm T)}\tau_{\rm sp}\right)\right] \times c\ , \tag{27}$$

where c is the fluorescence contrast of the optically detected magnetic resonance which for the NV ensemble $c = 0.04$.

Hence we may relate the difference in PL to the external component of the relaxation rate via:

$$\Gamma_1^{(\mathrm{T})} = -\frac{1}{c\,\tau_{\mathrm{sp}}} \log_e \left(1 - \frac{\Delta_{\mathrm{PL}}}{\mathrm{PL}_0}\right). \quad (28)$$

In cases where the environmental influence is very weak (small $\Gamma_1^{(\mathrm{T})}$) the change in PL, and hence the contrast $\left(\frac{\Delta_{\mathrm{PL}}}{PL_0}\right)$, will be small, hence we may use the formula $\log_e(1 + x) \sim x$, for small x

$$\Gamma_1^{(\mathrm{T})} = -\frac{1}{c\,\tau_{\mathrm{sp}}} \log_e \left(1 - \frac{\Delta_{\mathrm{PL}}}{\mathrm{PL}_0}\right),$$

$$\sim \frac{1}{c\,\tau_{\mathrm{sp}}} \frac{\Delta_{\mathrm{PL}}}{\mathrm{PL}_0} \quad (28)$$

Supplementary References:

1. Doherty MW, Manson NB, Delaney P, Jelezko F, Wrachtrup J, Hollenberg LCL. The nitrogen-vacancy colour centre in diamond. *Physics Reports* **528**, 1-45 (2013).
2. Hall LT, Cole JH, Hollenberg LCL. Analytic solutions to the central-spin problem for nitrogen-vacancy centers in diamond. *Phys Rev B* **90**, 075201 (2014).
3. McGuinness LP*, et al.* Ambient nanoscale sensing with single spins using quantum decoherence. *New J Phys* **15**, 073042 (2013).
4. Lewis WB, Alei M, Morgan LO. Magnetic Resonance Studies on Copper(II) Complex Ions in Solution. I. Temperature Dependences of the 17O NMR and Copper(II) EPR Linewidths of Cu(H2O)62+. *The Journal of Chemical Physics* **44**, 2409-2417 (1966).
5. Hall LT*, et al.* Detection of nanoscale electron spin resonance spectra demonstrated using nitrogen-vacancy centre probes in diamond. *Nat Commun* **7**, 10211 (2016).
6. Epstein RJ, Mendoza FM, Kato YK, Awschalom DD. Anisotropic interactions of a single spin and dark-spin spectroscopy in diamond. *Nat Phys* **1**, 94-98 (2005).
7. Glenn DR*, et al.* Single-cell magnetic imaging using a quantum diamond microscope. *Nat Meth* **12**, 736-738 (2015).
8. Gould M, Barbour RJ, Thomas N, Arami H, Krishnan KM, Fu K-MC. Room-temperature detection of a single 19 nm super-paramagnetic nanoparticle with an imaging magnetometer. *Appl Phys Lett* **105**, 072406 (2014).
